# Growth, immune and viral responses in HIV infected African children receiving highly active antiretroviral therapy: a prospective cohort study

**DOI:** 10.1186/1471-2431-10-56

**Published:** 2010-08-06

**Authors:** Philippa M Musoke, Peter Mudiope, Linda N Barlow-Mosha, Patrick Ajuna, Danstan Bagenda, Michael M Mubiru, Thorkild Tylleskar, Mary G Fowler

**Affiliations:** 1 Department of Paediatrics and Child Health, Makerere University, Kampala, Uganda; 2 Makerere University-Johns Hopkins University Research Collaboration, Kampala, Uganda; 3 School of Public Health, Makerere University, Kampala, Uganda; 4 Center for International Health, University of Bergen, Norway; 5 Johns Hopkins University, Baltimore MD, USA

## Abstract

**Background:**

Scale up of paediatric antiretroviral therapy in resource limited settings continues despite limited access to routine laboratory monitoring. We documented the weight and height responses in HIV infected Ugandan children on highly active antiretroviral therapy and determined clinical factors associated with successful treatment outcomes.

**Methods:**

A prospective cohort of HIV infected children were initiated on HAART and followed for 48 weeks. Body mass index for age z scores(BAZ), weight and height-for-age z scores (WAZ & HAZ) were calculated: CD4 cell % and HIV-1 RNA were measured at baseline and every 12 weeks. Treatment outcomes were classified according to; both virological and immunological success (VS/IS), virological failure and immunological success (VF/IS). virological success and immunological failure (VS/IF) and both virological and immunological failure (VF/IF).

**Results:**

From March 2004 until May 2006, 124 HIV infected children were initiated on HAART. The median age (IQR) was 5.0 years (2.1 - 7.0) and 49% (61/124) were female. The median [95% confidence interval (CI)] BAZ, WAZ and HAZ at baseline were 0.29 (-2.9, -1.2), -1.2 (-2.1, -0.5) and -2.06 (-2.9, -1.2) respectively. Baseline median CD4 cell % and log10 HIV-1 RNA were; 11.8% (7.5-18.0) and 5.6 (5.2-5.8) copies/ml. By 48 weeks, mean WAZ and HAZ in the VF/IS group, which was younger, increased from - 0.98 (SD 1.7) to + 1.22 (SD 1.2) and from -1.99 (1.7) to + 0.76 (2.4) respectively. Mean increase in WAZ and HAZ in the VS/IF group, an older group was modest, from -1.84 (1.3) to - 0.41 (1.2) and -2.25 (1.2) to -1.16 (1.3) respectively. Baseline CD4 cell % [OR 6.97 95% CI (2.6 -18.6)], age [OR 4.6 95% CI (1.14 -19.1)] and WHO clinical stage [OR 3.5 95%CI (1.05 -12.7)] were associated with successful treatment outcome.

**Conclusions:**

HIV infected Ugandan children demonstrated a robust increase in height and weight z scores during the first 48 weeks of HAART, including those who failed to completely suppress virus. Older children initiating HAART with severe immune suppression were less likely to achieve a successful treatment outcome. These data emphasize the importance of initiating HAART early to ensure adequate immune and growth responses.

## Background

Growth failure is a well recognized complication of HIV infection in children which can present as stunting, weight loss, failure to thrive and severe acute malnutrition which respond poorly to nutritional rehabilitation [1-3]. The mechanism for this growth failure is complex and multifactorial, including inadequate caloric intake, gastrointestinal infestations, opportunistic infections and HIV enteropathy [4,5]. Abnormal resting energy expenditure and endocrine abnormalities may also contribute to the diminished growth noted in perinatally HIV infected children [6]. In Africa, HIV related nutritional deficiencies compound the already high back-ground rates of malnutrition found in young children [7]. In Uganda, 39% and 16% of all children under five years of age are stunted and underweight respectively [8]. Although there is a high prevalance of growth failure in HIV infected children from sub-Saharan Africa, recent studies have reported a growth response among children initiated on highly active antiretroviral therapy (HAART), including significant weight gain and increase in height after some delay [9-11].

HAART has become more widely available for children from resource-limited settings. The monitoring of antiretroviral treatment response in children and adults from sub-Saharan Africa remains mainly clinical with limited access to immunological and virological tests, except in the larger cities [12]. Therefore it is critical to demonstrate that basic growth parameters, which can be measured by primary level health care workers in resource limited settings, are associated with successful HIV treatment outcomes. Sustained increase in height and weight in children on HAART have been linked to successful virological response [13]. The objective of this study was to document the growth response to HAART in a cohort of HIV infected Ugandan children and to determine clinical factors including growth parameters associated with successful virologic and immunologic treatment outcome.

## Methods

The study design and methods for this cohort of HIV infected children initiating HAART in Makerere University-Johns Hopkins University Collaboration (MU-JHU Care Ltd) HIV Care Clinic are reported elsewhere [14]. In summary, we conducted a prospective cohort study of HIV infected Ugandan children initiated on highly active antiretroviral therapy (HAART) between March 2004 and May 2006. Response to HAART was monitored using clinical measurements including height and weight; and laboratory parameters including CD4 cell counts/percents and HIV-1 RNA. The study was approved by the Makerere University Research and Ethics Committee and the Uganda National Council for Science and Technology. Informed consent was obtained from parents/guardians before study specific procedures were performed.

### Study population

A total of 130 HIV-infected children who were eligible for antiretroviral therapy (ART) were enrolled into the ART program at Makerere University-Johns Hopkins University Research Collaboration (MU-JHU) Kampala, Uganda. HIV infected children between 6 months and 12 years of age were initiated on HAART using the 2003 WHO criteria for antiretroviral therapy in resource-limited settings [15]. For this analysis, 124 children had growth measurements available and 123 completed the 48 weeks of follow up. Seven children died during the study follow up but one of them died after 44 weeks of follow up and therefore had data for most of the study time points except for week 48.

All the children received a non-nucleoside reverse transcriptase inhibitor (NNRTI)-based adult fixed dose combination (FDC) antiretroviral drug Triomune (CIPLA, India), containing stavudine (d4T 30 or 40 mg), lamivudine (3TC 150 mg) and nevirapine (NVP 200 mg). Those children who weighed less than 10 kg at HAART initiation, were prescribed syrup formulations of the same antiretroviral drugs until they weighed > 10 kg, when they were switched to Triomune. The triomune tablet was dosed based on the weight of the child and the individual antiretroviral (ARV) dosage was estimated according to weight band dosing [16]. Therefore the children received 1/4, 1/2 or 1 tablet of Triomune depending on their weight band. Children who developed a rash due to NVP hypersensitivity (n = 2) had their Triomune discontinued and they were switched to an alternative regimen, d4T + 3TC + efavirenz (EFV). None of the children were switched to second line antiretroviral therapy during the 48 weeks of follow up.

### Procedures

Study visits were conducted at baseline and every week for the first month, every two weeks for the second month and then every four weeks until 48 weeks. At the routine follow-up visits, anthropometric measurements were recorded, a physical exam was performed as was clinical staging, based on the 2003 WHO staging for infants and children. At baseline and every 12 weeks, blood was collected for CD4 cell counts/percents and plasma HIV-1 RNA. Children also received follow up clinical care which included multivitamins, cotrimoxazole prophylaxis, and free medication for all acute illnesses. Caregivers and children, if age appropriate, received adherence counseling at each scheduled follow-up visit. Adherence monitoring was conducted at each follow-up visit and was based on pill counts and self-reports from caregivers. Caregivers were provided with a 30 day supply of the antiretroviral medication.

## Measurements

### Growth measurements

Heights and weights were recorded for each child at each routine follow-up visit. This was done by staff trained in carrying out accurate measurements; and standardized stadiometers and scales were used. The body mass index (BMI) was calculated from height and weight measures as defined by BMI equal to weight (kilograms)/height (meter^2^). Median weight-for-age z score (WAZ), median height-for-age z score (HAZ) and body mass index-for-age z score (BAZ) were calculated using the WHO-child growth standards [17]. A z score of 0 represents the p50 which is the median age/sex WHO based reference population. A z score of -1 indicates that the child's weight or height is 1 standard deviation (SD) below the median weight or height for the reference population.

### Laboratory measurements

Participants had blood drawn at baseline for measurement of complete blood cell count (CBC), CD4 cell count/percent, liver transaminases, renal function tests (creatinine) and plasma HIV-1 RNA. Repeat CD4 cell counts/percents and plasma HIV-1 RNA levels were done at 12, 24, 36 and 48 weeks after HAART initiation. All laboratory tests were performed at the MU-JHU Core Lab, Kampala Uganda, which is certified by the College of American Pathologists. The CD4 cell counts/percents were assessed by BD FACS Calibur instrument (Becton, Dickinson & Company). Plasma HIV RNA levels were measured using the Amplicor HIV-1 Monitor Test, version 1.5 Standard assay (Roche Company, Branchburg, New Jersey/USA). Plasma HIV-1 RNA levels >750,000 copies/ml were diluted and further assessed by dilution.

Laboratory based Definitions of Treatment Success used in this analyses: HIV-1 RNA levels that were <400 copies/ml were considered undetectable and a successful treatment outcome for the purpose of these analyses. *Virological treatment success *(VS) was defined as undetectable viral load (< 400 copies/ml) after 24 weeks of HAART and sustained through 48 weeks of follow up. *Immunological treatment success (IS) *was defined as achievement of a CD4 cell percent above the age cut off level for severe immunosuppression (CD4 cell percent; less than one year > 25%, one to five years > 20%, greater than five years > 15%) after 24 weeks of HAART and sustained throughout the follow up period.

### Statistical analysis

Descriptive statistics are presented as medians with inter-quartile ranges (IQR) and means with standard deviations (SD). Proportions of different baseline characteristics of study children and their respective 95% confidence intervals (CIs) are also provided. Chi square test p-values were used to compare baseline categorical variables. Kruskal Wallis p-values were used to compare medians of continuous variables at baseline. The primary endpoint for this analysis was achievement of a viral load less than 400 copies/ml at 24 weeks sustained through to 48 weeks, and achievement of CD4 cell count/percent above level of immune suppression for age. The children were classified into treatment outcome groups: virological and immunological success (VS/IS), virological success and immunological failure (VS/IF), virological failure and immunological success (VF/IS) and both virological and immunological failure (VF/IF). Mean WAZ and HAZ scores at the different time points of follow up after HAART initiation were compared using ANOVA and Scheffe's multiple comparison test. Factors associated with treatment success were analyzed using multinomial logistic regression and presented as univariate and multivariate analyses. In order to establish whether growth in children predicts virological and immunological treatment outcome after 48 weeks of HAART we conducted a logistic regression model for the different baseline z-scores and slope in the first 24 weeks. In this model, we categorized virological and immunological outcome as 1 (virological/immunological success) and the other treatment outcome groups as 0. The utility of the WAZ, HAZ or growth velocity as predictors of successful treatment outcome was determined by calculating the sensitivity, specificity and ROC curves. Generalized estimating equations (GEE) were used to analyze weight and height velocity for different age groups during the 48 weeks of follow-up adjusting for various baseline factors. All statistical analyses were assessed for statistical significance at the p < 0.05 alpha level. All statistical analyses were performed using STATA Version10 (Copyright 1984-2007 Statistics/Data Analysis StataCorp 4905 Lakeway Drive, College Station, TX 77845 USA).

## Results

### Study population

The baseline characteristics of the 124 HIV infected children enrolled into the study and initiated on HAART are presented in Table 1. The median age was 5.0 years (IQR 2.1 -7.0) and 49% (61/124) were female. Only six (4%) children were below 12 months of age and the majority, 65% (80/124) were over 3 years of age. The children were more likely to be stunted than wasted with a median HAZ of -2.0 (IQR -2.9, -1.2) and WAZ of -1.2 (IQR -2.1, - 0.5) at baseline. The median CD4 cell percent and log10 HIV RNA were 11.75% (IQR 7.5 - 18.0) and 5.55 (5.2 - 5.8) copies/ml respectively. All the children were ART naïve at enrollment and 95% (123/130) completed 48 weeks of follow up. Seven children died during the study follow up; the causes of death included presumed pneumocystis carinii pneumonia, cerebral toxoplasmosis, HIV nephropathy, severe anaemia and diarrhoea. None of the deaths were related to the antiretroviral drugs. All the children were on their initial HAART regimen at the end of the 48 weeks. After HAART initiation the children had fewer sick visits during weeks 24 - 48 when compared to the initial 24 weeks on therapy (data not shown). On the basis of treatment outcome there were 80 (65%) VS/IS, 27 (22%) VS/IF, 10 (8%) VF/IS and 7 (5%) VF/IF children, in the respective groups [Table 1]. The baseline characteristics of each group were similar except for their baseline; age, WAZ category, BAZ, log HIV-1 RNA and CD4 cell %. The VF/IS treatment outcome category were the youngest with a median age of 1.7 years (IQR 0.9-2.8) and had the highest median viral load of 5.88 copies/ml (IQR 5.7 - 5.9). The immunological failure groups (VS/IF and VF/IF) both had baseline median CD4 cell percent below 10% at study entry and they were significantly different from the immunological success groups (VS/IS & VF/IS) (p = 0.0001).

**Table 1:** Baseline characteristics of the children according to antiretroviral treatment outcome group

Characteristic	All	VS/IS**	VS/IF	VF/IS	VF/IF	*P*
Number (%)	124	80 (65)	27 (22)	10 (8)	7 (5)	
Sex n(%)						
Male	63 (51)	40 (50)	13 (48)	5 (50)	5 (71)	
Female	61 (49)	40 (50)	14 (52)	5 (50)	2 (29)	0.73†
Age (years)						
median (IQR)	5.0 (2.1- 7.0)	4.7 (2.0 -8.6)	7.0 (5.4-9.8)	1.7 (0.9 - 2.8)	4.08(1.3 -6.6)	0.0001*^a^
BAZ¥						
Median (IQR)	0.29 (-2.9, -1.2)	0.42 (-0.8, 0.9)	-0.42 (-1.7, 0.4)	0.44 (0.2, 1.3)	0.37 (0.3, 1.0)	0.01*^ a^
WAZ¥						
median (IQR)‡	-1.2 (-2.1, - 0.5)	-1.13 (-1.9, - 0.2)	-2.10 (-2.9,-1.2)	-1.33 (-2.1, -0.6)	-0.90 (-1.1, -0.6)	0.06^a^
HAZ¥						
median (IQR)	-2.0 (-2.9, -1.2)	-1.95 (-2.6, -1.1)	-2.39 (-3.2, -1.42)	- 2.55 (-3.7, -1.3)	-1.99 (-3.2, -1.5)	0.18^a^
#WHO stage n (%)						
Stage1	23 (19)	14(18)	6 (22)	2 (20)	1(14)	
Stage 2	63 (51)	37(46)	17 (63)	5 (50)	4(57)	
Stage 3	38 (30)	29(36)	4 (15)	3 (30)	2(29)	0.61
WAZ category‡						
WAZ >-2	81 (72)	58 (77)	10 (48)	7 (70)	6 (100)	
WAZ <=-2	31 (28)	17 (23)	11 (52)	3 (30)	0 (0)	0.02*
HAZ category						
HAZ >-2	62 (50)	42 (53)	12 (44)	4 (40)	4 (57)	
HAZ <=-2	62 (50)	38 (47)	15 (55)	6 (60)	3 (43)	0.78
BAZ category						
BAZ >-2	117 (94)	75 (94)	25 (93)	10 (100)	7(100)	
BAZ <=-2	7 (6)	5 (6)	2 (7)	0 (0)	0 (0)	0. 75

Log_10 _HIV-1 RNA						
median (IQR)	5.55 (5.2 - 5.8)	5.54 (5.2 - 5.9)	5.36 (4.9 - 5.7)	5.88 (5.7 - 5.9)	5.76 (5.6 - 6.2)	0.007*^ a^
CD4 cell %						
median (IQR)§	11.75 (7.5 - 18.0)	14.7 (10.3 -19.7)	7.2 (3.3 - 9.0)	14.6 (8.4 - 21.0)	5 (3.8 -11.1)	0.0001*^ a^
CD4 cell % category						
n (%)						
≤ 5%	19 (16)	6 (8)	8 (31)	1 (10)	4 (57)	
> 5% - ≤ 10%	30 (25)	13 (17)	13 (50)	3 (30)	1 (14)	
> 10%	71 (59)	58 (75)	5 (19)	6 (60)	2 (29)	< .0001*

^a ^Kruskal Wallis tests for p values and others based on Pearson Chi-squared test *Statistically significant at the 0.05 alpha level ‡n = 112 because 12 children >10 yrs #according to WHO paediatric clinical staging 2003 §4 missing CD4 cell percents at baseline. ¥BAZ = basal metabolic rate z score, ¥WAZ = weight for age z score, ¥HAZ = height for age z score. **VS = virological success, IS = immunological success, VF = virological failure, IF = immunological failure.

### Changes in growth parameters of children on HAART according to treatment outcome group

**Figure 1:**
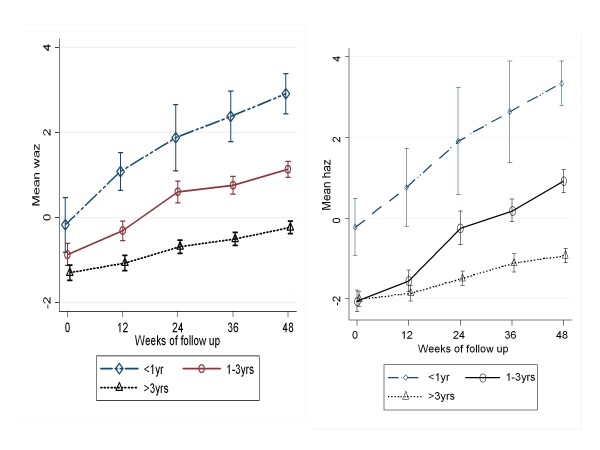
**Mean WAZ (*left*) and HAZ(*right*) scores from baseline to 48 weeks in the VS/IS treatment outcome group according to age category**.

Mean changes in WAZ and HAZ scores during the 48 weeks of HAART therapy by treatment outcome groups are presented in Figure 1. The BMI z scores did not change significantly over the follow up period. There were no statistically significant differences in BMI z scores between the treatment failures and the treatment success groups at any of the time points (data not shown). By the end of the study all the treatment outcome groups had a significant increase in mean growth z scores regardless of their virological and immunological treatment outcome. The overall mean WAZ and HAZ scores increased from -1.14 (SD) and -2.06 (SD) at baseline to + 0.6 (SD) and -0.41 (SD) respectively by 48 weeks (p = 0.001).

The VS/IS and VF/IS groups both had significant improvements in mean HAZ and WAZ scores on therapy, suggesting that complete viral suppression is not a requirement for an initial increase in weight and height (Figure 1). The VF/IS category (which comprised of comparatively younger children) had a more robust increase in mean growth z scores with a WAZ increasing from - 0.98 (SD 1.7) to + 1.22 (SD 1.5) and HAZ from - 1.99 (SD1.7) to 0.76 (SD 2.4) (Table 2 and 3). Unlike the other treatment outcome groups, the VS/IF group had a slow and modest increase in HAZ and did not achieve a mean HAZ of 0 by the end of the 48 week follow up (Figure 1). The VF/IF group had greater than 1 SD unit increase in WAZ and HAZ, without complete suppression of virus or restoration of CD4 cells. However, this small group (n = 7) experienced a log drop in median viral load (mean 5.8 to 4.8) and a significant increase in median CD4 cell percent (5.0 to 17.4) between baseline and 48 weeks.

**Table 2:** Mean WAZ values at baseline and 24 and 48 weeks after HAART initiation

*Treatment Outcome group*	*Baseline(O)*	*WAZ values 24 wks*	*48 wks*	*P*
VS/IS (n = 75)#				
WAZ mean (SD)	-1.07 (1.3)	-0.05 (1.4)	0.50 (1.2)	< 0.0001*
VS/IF (n = 21)				
WAZ mean (SD)	-1.84 (1.3)	-1.02 (1.3)	- 0.41 (1.2)	0.002
VF/IS (n = 10)				
WAZ mean(SD)	-0.98 (1.7)	0.44 (1.5)	1.22 (1.5)	0.013
VF/IF (n = 6)				
WAZ mean (SD)	-0.70 (0.63)	0.37 (0.76)	0.97 (0.8)	0.004

†12 missing WAZ values were > 10 yrs *ANOVA, statistically significant at the 0.05 alpha level. #n = 75, 71 & 69 at 0, 24 and 48 weeks respectively. WAZ = Weight for Age z score. VS = virological success, VF = virological failure, VF = virological failure and IF = immunological failure.

**Table 3:** Mean HAZ values at baseline and 24 and 48 weeks after HAART initiation

Treatment Outcome group	Baseline(0)	HAZ values 24 wks	48 wks	P
VS/IS (n = 80)				
HAZ mean (SD)	-1.95 (1.4)	-0.88 (1.9)	0.005 (1.8)	< 0.0001*
VS/IF (n = 27)				
HAZ mean (SD)	-2.25 (1.2)	-1.88 (1.2)	- 1.16 (1.3)	0.005
VF/IS (n = 10)				
HAZ mean(SD)	-1.99 (1.7)	- 0.76 (1.5)	0.59 (2.4)	0.031
VF/IF (n = 7)				
HAZ mean (SD)	- 2.03 (1.3)	-1.13 (1.0)	- 0.07 (1.1)	0.018

*ANOVA, statistically significant at the 0.05 alpha level. # n = 80, 75 & 73 at 0, 24 and 48 weeks respectively. HAZ = Height for age z score. VS = virological success, VF = virological failure, VF = virological failure and IF = immunological failure.

**Figure 2:**
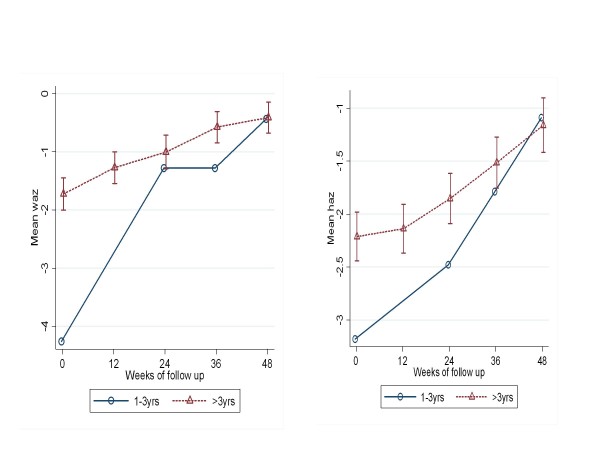
**Mean WAZ(*left*) and HAZ(*right*) scores from baseline to 48 weeks in the VS/IF treatment outcome group according to age category**.

When the treatment outcome groups were categorized by age, the VS/IF and VF/IF groups did not include any children less than one year of age, indicating than none of the infants had experienced immunological failure by 48 weeks on HAART [Figure 2, 4]. All children had a significant mean increase in WAZ and HAZ but the 1-3 year olds (p < 0.0001) and the > 3 year olds (p < 0.0001) had a less robust increase in WAZ and HAZ when compared to infants <1 year of age [Figure 1, 4].

**Figure 3:**
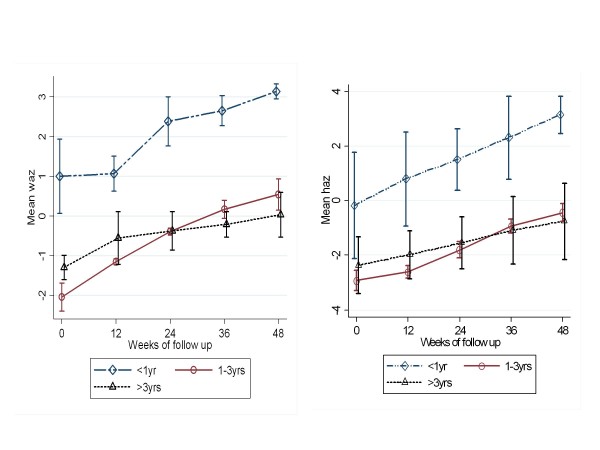
**Mean WAZ(*left*) and HAZ(*right*) scores from baseline to 48 weeks in the VF/IS treatment outcome group according to age category**.

**Figure 4:**
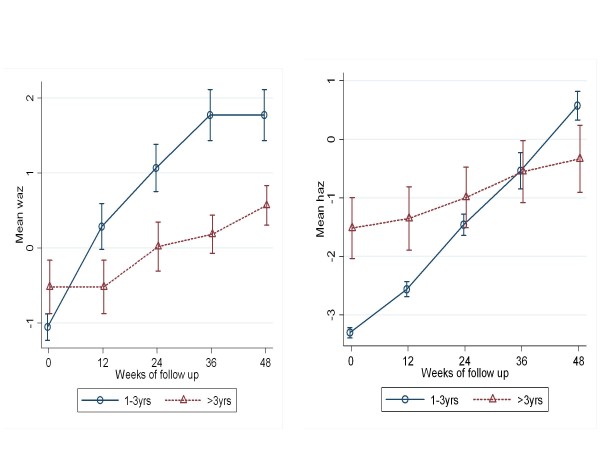
**Mean WAZ(*left*) and HAZ(*right*) scores from baseline to 48 weeks in the VF/IF treatment outcome group according to age category**.

### Factors associated with treatment outcomes

When the baseline characteristics were placed into a univariate model for association with the successful treatment outcome group (VS/IS) compared to the other treatment outcome groups combined, only CD4 cell % at baseline was significantly associated with successful treatment outcome [Table 4]. The children with CD4 cells > 10% at HAART initiation were seven times more likely to have a successful treatment outcome (VS/IS) when compared to those children with a CD4 cell percent < 10%. The other factors, including age, sex, HIV RNA, WAZ and HAZ were not significantly associated with successful treatment outcome. In a multivariate model age and WHO clinical stage were also associated with successful treatment outcome after adjusting for sex, HIV RNA, CD4 cell %, WAZ and HAZ [Table 4]. The mean HAZ and WAZ slopes from baseline to 48 weeks were indicatively similar in all the groups except for the VS/IF group, which was older, where the slopes for WAZ and HAZ were much less than the other treatment outcome groups [Figure 2]. Weight and height velocity were not significantly associated with treatment outcome when age, baseline CD4 cell % and WHO clinical stage were included in the GEE model.

**Table 4:** Baseline factors associated with successful treatment outcome (VS/IS)

*Baseline characteristic*	*Unadjusted OR (95% CI)*	*Adjusted OR (95% CI) *†
Age > 18 months (vs < 18 mths)	1.07 (0.39 - 2.96)	4.6 (1.14 -19.1)*
Sex Male (vs Female)	0.91 (0.44 - 1.91)	0.71 (0.27 - 1.85)
CD4 cell % >10% (vs ≤ 10%)	7.04 (3.07 - 16.19)**	6.97 (2.6 - 18.6)**
HIV-1 RNA (copies/ml) < 750,000 (vs ≥ 750,000)	1.95 (0.51 - 7.51)	1.42 (0.29 - 6.98)
WHO stage 1&2 (vs 3)	2.21 (0.93 - 5.24)	3.50 (1.05 - 12.7)*
WAZ ≤ -2 (vs ≥ -2)	0.48 (0.20 -1.13)	0.50 (0.14 -1.78)
HAZ ≤ -2 (vs > -2)	0.79 (0.38 - 1.66)	1.19 (0.39 - 3.65)

†Adjusted for age, sex, CD4 cell %, viral load, WHO stage, WAZ and HAZ. **p < 0.0001 *p = < 0.05. HAZ = Height for age z score, WAZ = weight for age z score and WHO = World Health Organization.

In a multinomial logistic regression, older age >18 months (p < 0.001), lower (<10%) baseline CD4 cell % (p = 0.001) and lower (<-2 SD) baseline HAZ (p < 0.001) were significantly associated with immunological failure alone (VS/IF), while lower age < 18 months (p = 0.027) was associated with virological failure alone (VF/IS); and lower CD4 cell % (p = 0.014) was associated with an observation of a both a virological and immunological failure(VF/IF) at 48 weeks. Age had the greatest predictive probability (44%) for observing both an immunologic and virologic success.

### Viral and immune response to HAART according to treatment outcome groups

Eighty six percent (107/124) of the children had undetectable viral load (< 400 copies/ml) and 73% (90/124) had immune restoration after 48 weeks of HAART. The proportion of children less than one year, one to three years and over three years of age with VS at 48 weeks were 50% (3/6), 82% (34/38) and 90% (68/73) respectively. The proportion of children with immunological success (IS) in the same age groups were 83% (5/6), 97% (37/38) and 89% (65/73) respectively. The one child under one year of age (1/6) who did not achieve immunological success at 48 weeks had a CD4 cell percent of 24%. The mean CD4 cell percent after 48 weeks of HAART was significantly different for the 4 categories: 32.4% (SD 7.0) in the VS/IS, 18% (SD 4.9) in the VS/IF, 32.2% (SD 4.9) in the VF/IS, 17.5% (SD 4.5) in the VF/IF group (p < 0.001). Twenty four weeks after HAART initiation, the mean log10 HIV RNA in the VS/IS, VS/IF, VF/IS and VF/IF was also different: 2.6 (SD 0.37), 2.7 (SD 0.13), 4.0 (SD 0.80) and 4.8 (SD 0.79) respectively (p < 0.01). By definition, all the children in the VS/IS and VS/IF groups had HIV-1 RNA < 400 copies/ml at 24, 36 and 48 weeks on HAART; none of the children in the VF/IS and VF/IF groups had a viral load < 400 copies/ml at the same time points.

### Utility of growth parameters for predicting successful treatment outcomes

Used by themselves, the baseline WAZ, HAZ and 24 week WAZ and HAZ, and first 24 week WAZ and HAZ velocity showed modest to low sensitivity (< 63%) and specificity (< 42%) (data not shown) for predicting successful treatment outcome. However, when they were used together in a model to predict success or failure at a cut-off of model probability >0.5, there was increased sensitivity of 87.3%, but very low specificity of 27.0%.

## Discussion

Similar to other studies from Africa and other resource limited settings, HIV infected children in this study experienced a significant increase in height and weight after initiation of NNRTI based HAART [18-24]. The younger VF/IS group experienced a robust growth response despite failing to completely suppress virus on therapy. Our findings are similar to those of Ghaffari et al who found that the children on HAART with immunological treatment success, and with or without complete viral suppression had similar increases in weight and height z scores at 48 and 96 weeks post therapy [25]. The authors postulate that although the virus may still replicate post therapy, its effect on growth may be less deleterious. Our study findings concur with the above observation and suggests that complete viral suppression may not be a requirement for the initial growth response in children on HAART [26] as long as there is a robust immune response. Children have high lymphoproliferative responses compared to adults which may help explain these findings. In contrast, Verweel et al found that the children on HAART who were viral responders had significant increases in height and weight while the non responders did not [13]. Guillen et al also found that children with complete viral suppression had more significant increases in height and weight when compared to children with inadequate viral suppression [27]. The differences noted in these two studies could be related to their small sample size, older age of the children, longer follow up period, or due to most of their study children being on second line therapy at the time of evaluation. In comparison, our cohort were all ART naïve, had a high rate of adherence to the antiretroviral fixed dose combination and the VF/IF group also had an initial modest response to therapy. However, other studies have also documented the significant increase in growth velocity while on HAART, without complete viral suppression [2,10,26]. Similar to our findings these studies also found no significant change in BMI on therapy and suggest that unlike adults children on therapy increase in both height and weight and therefore maintain the same BMI.

Older infected children in resource limited settings are usually long term survivors with severe immunosuppression and are less likely to have a rapid increase in height on HAART [26]. In our study, the older VS/IF group had the poorest growth response to HAART suggesting that immune reconstitution may be more critical than complete viral suppression for initial growth response in children [25]. When compared to the other treatment outcome groups in the study, these children had a higher baseline viral load, were more severely immunosuppressed and wasted and their growth response to therapy was modest. A longer follow-up may have provided more information on their potential for catch-up growth. However, considering findings from other studies with longer follow up, increases in height are less pronounced when HAART is initiated later [26,28]. Therefore, to ensure that HIV infected children reach their full height potential, HAART needs to be initiated early before irreversible stunting occurs. This requires early identification of HIV infected children and could be achieved through scale up of national early infant HIV diagnosis (EID) programs using dried blood spots (DBS)[29]. From our data, children who were not showing a significant increase in height or weight were also failing immunologically. Another study found that height growth velocity was a predictor of survival independent of viral load, age and CD4 cell count in HIV infected children on HAART [2]. In our cohort weight and height velocity over the 48 weeks did not predict virologic or immunologic treatment outcome. This may be related to the initial drop in viral load and modest increase in CD4 cell percent in most children leading to an initial growth response.

The children who initiated HAART with a low CD4 cell percent, regardless of age were less likely to have a robust increase in CD4 cells. It seems these children, in fact have lost their potential for 'good enough' immunological reconstitution and therefore they do not reach our threshold for 'immunological success' within the first 48 weeks on therapy. These findings are consistent with other studies where delay in initiation of HAART is more likely to lead to treatment failure in children and adults [19,20,28]. Our study further emphasize the importance of initiating HAART early in children so as to suppress virus, restore immune function and enable satisfactory somatic growth. It is critical to ensure that these initial growth responses in children from sub-Saharan Africa are sustained beyond the early years of therapy. Longer term follow up of children on HAART from Thailand, report younger age, higher baseline CD4 cell % and sustained viral suppression after 24 weeks as predictors of immune recovery at week 96 [28]. In addition, a study from Cote d'Ivoire reported improved survival and sustained viral suppression with good immune recovery in children on HAART for a median follow up of 36 months [30].

Our documentation of early growth responses on HAART, despite inadequate viral suppression in children from a resource limited setting is encouraging. However, the continued replication of virus on HAART leads to selection of resistant mutations which may limit future treatment options [20,31,32]. Children in resource limited settings have even more limited treatment options and remaining on a first line regimen with adequate immune recovery and adequate growth response may be more important than switching to second line therapy to ensure complete viral suppression [25]. Therefore it is important to develop strategies that ensure good adherence to the first line regimen and close clinical monitoring may be the better option for scaling up ART and improving survival of HIV infected children from resource limited settings. In the larger Ugandan population similar robust growth responses may not occur in all children initiating HAART because of the varying infectious diseases and socio-economic factors in the home.

There were certain limitations to our study including the small number of children in the failure groups and the lack of growth data for HIV negative control children which may have affected the results. Also, the follow-up duration was 48 weeks so we were unable to assess the long term impact of HAART on growth, despite inadequate viral suppression. However, the strength of this study is that it was a prospective cohort with longitudinal clinical and laboratory data collected consistently with no child lost to follow up, except for those who died. In addition, the children had high adherence levels to HAART and the majority experienced viral and immune treatment success with associated improvement in growth. Larger studies with longer follow up on therapy would be beneficial in documenting the sustained growth response of infected children and guiding future treatment strategies for children from resource limited settings.

## Conclusion

In a cohort of HIV infected Uganda children we demonstrated a significant increase in weight and height during treatment with HAART. The age at initiation of HAART determined the magnitude of the growth response with the more significant increases in weight and height response documented in the youngest age group (<1 year). Of note, those children who did not experience complete viral suppression on therapy but did show immunologic success also showed a marked improvement in growth parameters. Children who initiated therapy with severe immune suppression were less likely to restore immune function and only had modest growth response. These data emphasize the importance of initiating HAART early to ensure adequate immune and growth responses.

## Competing interests

The authors declare that they have no competing interests.

## Authors' contributions

PM contributed to the statistical analysis and writing of the manuscript, LBM contributed to the conduct of the study (study coordinator), collection of data, analysis and writing of the manuscript, PA contributed to collection of data and analysis, DB (senior statistician) contributed to study design, statistical analysis, data interpretation and writing of the manuscript, MM contributed to the statistical analysis and interpretation, TT contributed to the analysis, review and writing of the manuscript. MGF contributed to the writing and review of manuscript. *PMM(principal investigator) corresponding author, contributed to the study design and conduct, analysis and writing of the manuscript*. All authors read and approved the manuscript and agreed to the submission.

## Pre-publication history

The pre-publication history for this paper can be accessed here:

http://www.biomedcentral.com/1471-2431/10/56/prepub
